# Effects of *Lupinus luteus* on hepatic and renal extracellular compounds turnover under diabetes in rat

**DOI:** 10.1002/fsn3.3200

**Published:** 2022-12-27

**Authors:** Maha Sayari, Houcine Dab, Mounira Tlili, Lazhar Zourgui, Abdelmajid Khabir

**Affiliations:** ^1^ Laboratory of Biodiversity Molecules, Applications, (LR22ES02) Higher Institute of Applied Biology Medenine, University of Gabes Medenine Tunisia; ^2^ Laboratory of integrative Physiology, Faculty of Sciences of Bizerte University of Carthage Bizerte Tunisia; ^3^ Department of Pathology Habib Bourguiba Hospital Medenine Tunisia

**Keywords:** diabetes, ECM, kidney, liver, MMP, yellow lupin

## Abstract

Hepatic and renal extracellular matrix (ECM) turnover associated with diabetes and potential beneficial effects of yellow lupin extract (YLE) need further investigations. The aim of this study was to explore the effect of yellow *Lupinus luteus* extract (YLE) on renal and hepatic ECM under diabetes. Composition of YLE performed by LC‐ESI‐MS. Diabetes (DM) was induced in rats by alloxan (250 mg/kg, ip). Normal and diabetic rats received 100 mg/kg of YLE for 1 month. ECM was assessed by ELISA. Gelatinases and collagenases were analyzed by a colorimetric assay. Histology was performed on sections of liver and kidney. In the liver, diabetes increases collagen, laminin, and fibronectin contents, respectively, by 49% (*p* < .01), 56% (*p* < .01), and 67% (*p* < .05) compared to control rats. In the kidney, total collagen and laminin contents were increased by 91% (*p* < .01) and 35% (*p* < .01) in the DM group, while fibronectin content in diabetic animals and those treated with YLE remains similar to the control group. Collagenases and gelatinases activities were significantly increased by diabetes in liver and kidney. While YLE treatment abrogates diabetes‐enhanced MMPs activities in liver. In diabetic rats, the liver shows signs of diffuse dilatation of the sinusoid veins and steatosis. However, the liver of diabetic rats treated with yellow lupine extract showed a normal histological aspect similar to controls. Diabetes causes hepatic and renal ECM turnover. YLE can be useful to partially improve tissue disorders induced by diabetes.

## Graphical abstract

*Lupinus luteus* abrogate changes induced by diabetes in ECM turnover and at histological level. There is the first study to investigate effect of *L. luteus* on diabetes and on hepatic and renal ECM turnover.

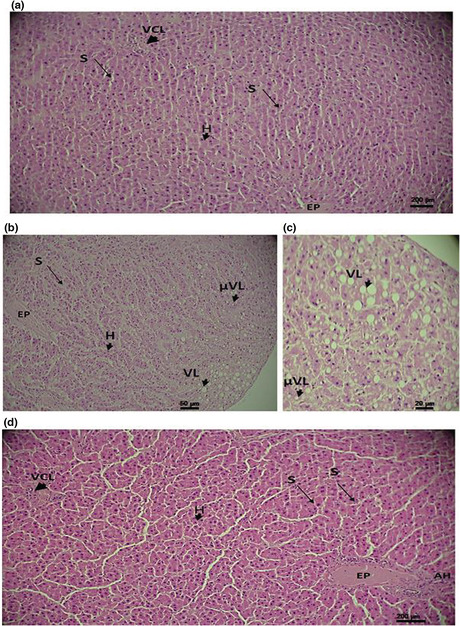

## 1 INTRODUCTION

Type 2 diabetes is a chronic metabolic disease, with an exponentially growing incidence rate. Thus, in Tunisia, type 2 diabetes prevalence was 19.6% in 2015 against 12% in 1997 (Saidi et al., 2015). This increase is mainly linked to lifestyle changes and eating habits of the population. Type 2 diabetes is characterized by decreased insulin secretion by pancreatic islet cells and insulin resistance by target cells and can lead to organic and functional damage to many organs (Wondmkun, 2020).

The extracellular matrix (ECM) is composed by several molecules such as collagen, elastin, laminin, and fibronectin which changes in development and in pathophysiological process as fibrosis, osteoarthritis (Sonbol, 2018). The ECM is tightly regulated to achieve a delicate balance between the synthesis of its constituents and their degradation by specialized enzymes such as matrix metalloproteinases (MMPs). Tissue remodeling is defined as any long‐term change in the ECM composition and level in tissue that allows adaptation and/or repair under normal or pathological conditions (Sonbol, 2018). In pathophysiological processes, an imbalance of proteinase/antiproteinase systems occurs, resulting in quantitative and qualitative alterations in matrix composition (Berg et al., 2019).

The Yellow Lupin (*Lupinus luteus*) is an annual plant reaching 80 cm in height and cultivated in Mediterranean regions (Parra‐González et al., 2012). Lupin was used in a variety of food applications such as to prepare bread for diabetic patients and also for animal feed (van de Noort, 2017). Several species of lupin have been studied for their therapeutic benefits and their antidiabetic effect such *Lupinus albus* and *Lupinus mutabilis* (Garmidolova et al., 2022). However, there are no previous studies interested in the effect of *L. luteus* on diabetes.

In this context, the aim of this study is to investigate the involvement of diabetes in hepatic and renal ECM remodeling and the potential beneficial effects of *L. luteus* extract.

## 2 MATERIALS AND METHODS

### 2.1 Preparation of yellow lupine extracts

*L. luteus* seeds were ground using a food processor. Fifteen gram of the powder was extracted in 100 ml of ethanol 50% solvent and stirred for 24 h at room temperature in dark. The mixture was filtered on filter paper to remove the solid particles and solvents were removed using a rotary evaporator.

### 2.2 Chromatography of phenolic acids and flavonoids by LC‐ESI‐MS

Separation of phenolic extracts, sugars, and vitamins was performed using LC‐ESI‐MS on Shimadzu UFLC XR system, equipped with SIL‐20AXR autosampler, SCL‐10A system controller, AC CTO‐20 column, LC‐20ADXR binary pump, and a 2020 quadrupole detection system. This instrument was equipped with a Discovery BIO Wide Pore C18 column (S250 × 4.0 mm id; 5 μm). The column temperature was set at 40°C and the injection volume was 5 μl with a flow rate of 0.5 ml/min. Water with 0.1% formic acid and methanol with 0.1% formic acid were used as mobile phases A and B, respectively. Samples were analyzed using a linear gradient programmed as follows: 0–14 min, 10–20% B; 14–27 min, 20%–55% B; 27–37 min, 55%–100% B; 37–45 min, 100% B; and 45–50 min, 10% B.

The conditions used for MS with the electrospray ionization (ESI) source were as follows: the dissolution line temperature was 280°C, the drying gas, nitrogen, was set at 15.0 L/min, and the nebulization gas flow rate was 1.50 L/min. LC ESI (−) MS [M‐H]–mass spectra were acquired by Labsolutions software. The quantification and identification of phenolic compounds present in different samples were obtained by comparison with the retention time and mass spectra of known standards tested under the same conditions.

### 2.3 Determination of the content of total nitrogenous matter, total sugars, and vitamins

Total nitrogen was determined by the Kjeldahl method. The experimental approach consisted of mixing 0.5 g of ground and homogenized lupine powder with 12 ml of concentrated sulfuric acid and 1 g of selenium catalyst in a macro‐Kjeldahl tube, which was then placed in the digester for at least 60 min at 450°C. After digestion, 50 ml of sodium hydroxide (35%) was added, and ammoniacal nitrogen was recuperated in a 4% boric acid solution. Obtained distillate was titrated with a hydrochloric acid solution HCL (0.1 N).

### 2.4 Animals

The animal protocols were approved by the University Animal Care and Use Committee of the University of Gabes, Tunisia, and were in accordance with the National Institutes of Health Guidelines for the Care and Use of Laboratory Animals.

Wistar rats were purchased from the Faculty of Sciences of Gabes, Tunisia. After an adaptation period of 2 weeks, all rats were maintained at the same conditions of 12‐h light/dark cycle, temperature (23°C), humidity (70%), and fed a commercial pellet diet and tap water ad libitum.

Rats were divided into four groups as follows:

DM group (*n* = 7): Diabetic rats that have received intraperitoneal injection of saline solution (0.9%) for 1 month.

YLE group (*n* = 7): Normal rats that have received intraperitoneal injection of yellow lupin extract (100 mg/kg) diluted in saline solution (0.9%) for 1 month.

DM + YLE group (*n* = 6): Diabetic rats treated with yellow lupin extract (100 mg/kg) diluted in saline solution (0.9%) for 1 month.

CTRL group: Normal rat group (*n* = 8) has received an intraperitoneal injection of saline solution (0.9%) for 1 month.

Diabetes was induced in rats according to previous protocol (Dab et al., 2022). Briefly, a single intraperitoneal dose of alloxan (250 mg/kg) from Sigma Chemical Co. (St Louis, MO, USA) dissolved in saline (0.9% NaCl) solution was used. Animals were injected after an 18‐h fasting period and received 10% of glucose solution 24 h following injection. Blood glucose level was monitored daily with an electronic glucometer (ACCU‐CHEK®, Roche, India) by performing a small puncture at the tail.

At the end of the various treatments, each rat was weighed and sacrificed by overdose of urethane. Blood was collected from the inferior vena cava, and the kidney and liver were removed, frozen immediately, and stored at −80 °C.

### 2.5 Assay of serum parameters

The levels of urea, creatinine, triglycerides, cholesterol, HDL, AST, and ALT were assessed by an electronic automatic BA400 analyzer (Biosystems S.A., Spain) at the Habib Bourguiba University Medical Center, Medenine (Tunisia).

### 2.6 Total protein extraction

Protein extraction from liver and kidney was performed according to a previous protocol (Dab et al., 2022). Organs were ground in a lysis buffer (0.25 M sucrose, 0.05 M Tris–HCL, 1 mM EDTA, pH 7.4). After 24 h of incubation at −20°C, the samples were centrifuged at 9600 × *g* for 15 min. The supernatant was removed and stored at −60°C until use. The protein concentrations were measured in the supernatants using bovine serum albumin as a standard.

### 2.7 Assay of total collagen, fibronectin, and laminin

The total collagen content in the samples as well as the level of fibronectin and laminin were determined by the commercial ELISA Kits Bio Vision (K218‐100), Abcam (ab108850), and LifeSpan (LS‐F6465), respectively, according to the manufacturer's protocol.

### 2.8 Assay of matrix metalloproteinase activity

The measurement of collagenase activity (MMP‐1, MMP‐8, and MMP‐13) is determined by a colorimetric method using a Bio vision kit and according to the manufacturer's instructions.

Gelatinase (MMP‐2 and MMP‐9) activity was assayed using the Gelatinase Assay Kit (Bio Vision, Cat. No.444–100) according to the manufacturer's instructions and the protocol of Chu et al. (2019).

### 2.9 Histological study

The histological study was carried out in the Department of Pathological Anatomy and Cytology of the Habib Bourguiba Hospital in Medenine. The liver and kidney were fixed in 10% formalin and stored at ambient temperatures. The organs are then cut into small fragments on a formalin absorbent table and placed in cassettes and then embedded in paraffin. The paraffin sections were cut into 5‐μm‐thick slices and stained with hematoxylin and eosin (H&E), respectively, for 1 to 5 min and 5–10 min. After that slides were immersed in water and then in a 95% alcohol bath and followed by light green dye which stains ECM for 1 min, the preparations were observed and micro‐photographed at 100×, 200×, and 400× at several levels under Leica microscope for reading by a pathologist. Representative images were taken using an optical microscope (Leica) equipped with a camera.

### 2.10 Statistical analysis

The results are expressed as mean ± standard deviation. An analysis of variance (ANOVA) was used for the comparison between the different treatments. Pairwise comparisons were performed using Tukey's HSD test by statistica software (statsoft France). A *p*‐value < .05 was considered statistically significant.

## 3 RESULTS

### 3.1 Polyphenols and flavonoids content and LC–MS analysis

Our results show that the extraction yields of yellow lupine are 6.52%, 23.18%, and 31.45% for the three used solvents: water, ethanol (96%), and ethanol 50%, respectively.

The assay of total polyphenols was carried out according to the Folin–Ciocalteu method. Our results, reported in Table 1, show that the polyphenol content is about four times higher after extraction in ethanol 50% (119.55 ± 9.43 mg EAG/g) than in ethanol 96% (34.79 ± 3.26 mg EAG/g).

**TABLE 1:** Contents of total polyphenols (mg EAG/g) and flavonoids (mg EC/g) of *Lupinus luteus* extracts in ethanol 96%, ethanol (50%), and water

Solvents			
	Ethanol (96%)	Ethanol (50%)	Water
Total polyphenols (mg EAG/g extract)	34.79 ± 3.26	119.55 ± 9.43	26.56 ± 2.98
Flavonoids (mg EC/g extract)	53.97 ± 4.24	9.04 ± 0.5	7.25 ± 0.4

*Note*: The results are expressed as mean **±** standard deviation.

The flavonoid contents were similar in water and ethanol 50% (7.25 ± 0.4 mg EC/g and 9.04 ± 0.5 mg EC/g, respectively). However, the ethanol 96% extract contains the highest flavonoid contents (53.97 ± 4.24 mg EC/g) (Table 1).

The phenolic compounds in the extracts obtained were analyzed by liquid‐phase chromatography coupled with a mass spectrometry detector. The number of phenolic compounds identified are 4, 7, and 9, respectively, after extraction with water, ethanol 96%, and ethanol 50%. The concentration, in ppm, of the various compounds identified as well as their retention times are presented in Table 2 .

**TABLE 2:** List of compounds from the water, ethanol 96%, and ethanol 50% extract of *Lupinus luteus* seeds identified by LC‐ESI‐MS.

Compound name		Aqueous extraction		Ethanol 96% extract		Ethanol 50% extract	
		Retention time	Concentration (ppm)	Retention time	Concentration (ppm)	Retention time	Concentration (ppm)
1	Quinic acid	2,221	5,084,146	1,605	22,718	1,761	15,656,978
2	Cynarin	1,588	68,432	1,536	22,288	1,605	139,607
3	Gallic acid	1,605	5,433	1,625	1,371	1,935	0,205
4	Ferulic acid	19,444	3,236			19,471	7,95
5	Aphegenin‐7‐o‐glucoside			24,667	4,373	24,736	24,185
6	Quercetin					29,392	6,056
7	Naringinin			31,398	14,027	31,441	39,673
8	Apigenin			3,163	11,469	31,688	24,878
9	Acacetin			37,833	29,962	37,821	18,506
	Total phenol acids		5,092,815		24,089		15,665,133
	Total Flavonoids		68,432		82,119		252,905

### 3.2 Nitrogen, total sugars, and vitamins content

Total nitrogen is determined by the Kjeldahl method. The nitrogen content is estimated at 58.22% ± 5.96%. Analysis of the water‐soluble vitamin composition showed that yellow lupine contains two water‐soluble vitamins, retinol and k1. *L. luteus* seeds exhibited low levels of the following total sugars: fructose, glucose, sucrose, palatines, maltose, and lactose (Table 3).

**TABLE 3:** Water‐soluble vitamin content in yellow lupin extract

Vitamin name	Retention time	Concentration in ppm
Retinol	3.037	521.009
K1	16.237	117.157

### 3.3 Fasting blood glucose level

Blood glucose monitoring was carried out throughout the experiment with rats belonging to the four groups; firstly, to validate diabetes model, and secondly, to explore the effect of lupin extract on the blood glucose level. We showed that fasting glucose level at the beginning of the study was 121.25 ± 2.5 mg/dl. This level remained constant in the CTRL group until the end of the study period (122.25 ± 4.42 mg/dl). On the other hand, alloxan single injection caused a significant increase (*p* < .01) in fasting blood glucose from the first 3 days (311.77 ± 18.61 mg/dl). In addition, the average fasting blood glucose of diabetic rats at the end of the experiment was 325.44 ± 199.19 mg/dl (Table 4).

**TABLE 4:** Changes in biochemical serum parameters from control and treated rats

	Fasting blood glucose (mg/dl)	TC (mmol/l)	TG (mmol/l)	HDL (mmol/l)	LDL (mmol/l)	AST (U/l)	ALT (U/l)	Urea (mmol/l)	Creatinine (μmol/)	CRP (mg/l)
CTRL	121.25 ± 2.5	1.12 ± 0.08	2.15 ± 0.05	0.02 ± 0.01	0.17 ± 0.08	45.66 ± 16.80	15 ± 5.29	7.39 ± 1.42	37.46 ± 2.51	6.77 ± 1.25
YLE	119.38 ± 4.22	1.16 ± 0.09	2.05 ± 0.23	0.03 ± 0.00	0.19 ± 0.19	44.50 ± 4.95	20 ± 2.82	6.17 ± 0.31	36.05 ± 1.62	5.50 ± 0.99
DM	311.77 ± 18.61**	1.47 ± 0.08*	2.34 ± 0.11	0.09 ± 0.02	0.42 ± 0.35*	107.12 ± 10.67**	33.12 ± 5.84**	14.18 ± 2.91**	55.70 ± 4.54*	10.07 ± 1.75*
DM + YLE	203 ± 6.065**	1.37 ± 0.05*≠	2.15 ± 0.11≠	0.07 ± 0.01	0.32 ± 0.12*	91 ± 5.65*≠	29 ± 2.82**	13.12 ± 0.51**	52.45 ± 0.77*	9.75 ± 0.63*≠

*Note*: Data are mean ± SEM (*n* = 8 for each group). *p < .05, **p < .01 vs. CRTL, ≠*p* < .05 vs. DM.

Abbreviations: ALT, Alanine aminotransferase; AST, Aspartate aminotransferase; CRP, C‐reactive protein; CTRL, control group; DM + YLE, Diabetic rats treated with 100 mg/kg of ethanol 50% extract of yellow lupine for 1 month; DM, Diabetic rats receiving injection of alloxan 250 mg/kg, ip; HDL, high‐density lipoprotein; LDL, low‐density lipoprotein; TC, total cholesterol; TG, triglycerides; YLE, Control rats treated with 100 mg/kg of yellow lupine extract.

In DM + YLE group, the blood glucose level before treatment with *L. luteus* extract was 366 ± 33.94 mg/dl. This value was significantly reduced to half (*p* < .05) at the end of treatment with the yellow lupine extract. On the other hand, the blood glucose of rats treated only with the yellow lupine extract remained constant and similar to the control group, throughout the experimental period.

Our results confirm the success of the diabetes model for the two groups DM and DM + YLE. Moreover, dasting blood glucose increases for the DM group. Moreover, we show a tendency for the glycemia of the DM + YLE group to decrease at the end of the experiments.

### 3.4 Serum parameters level

The biochemical parameters relating to liver (ASAT and ALAT) and renal function (urea and creatinine), lipid profile (total cholesterol, HDL, and triglycerides), and CRP were measured.

Analysis of lipid profile parameters showed that cholesterol levels increased in diabetic rats by approximately 31% (*p* < .05) compared to controls. Furthermore, the total cholesterol level of rats from the diabetic group which received yellow lupine extract decreased by 7% compared to the DM group. Our results show that the triglyceride level in the DM group increased by 8% compared to the controls.

Diabetes significantly increases the levels of the two transaminases by 134% and 120%, respectively, for AST and ALT (*p* < .05). In addition, the association of diabetes and treatment with yellow lupine extract decreases the levels of both transaminases to levels statistically similar to controls.

Serum creatinine level was increased by 48% (*p* < .05) in the diabetic rats compared to the controls. On the other hand, the value of creatinine and urea in the YLE group was statistically similar to those of the control rats.

The uremia assay shows similar results to those of creatinine. Indeed, uremia increased by approximately two times (*p* < .05) in the DM group compared to the CTRL group. On the other hand, the extract of *L. luteus* causes a slight decrease in uremia and creatinine compared to the DM group but remains nonsignificant. In the YLE group, serum creatinine and uremia are statistically similar to those of control rats.

Our results show that diabetes increases the level of inflammation markers CRP by 48% in the DM group compared to controls. Lupin extract decreases slightly CRP level compared to the DM group (Table 4).

### 3.5 ECM compound level

**FIGURE 1:**
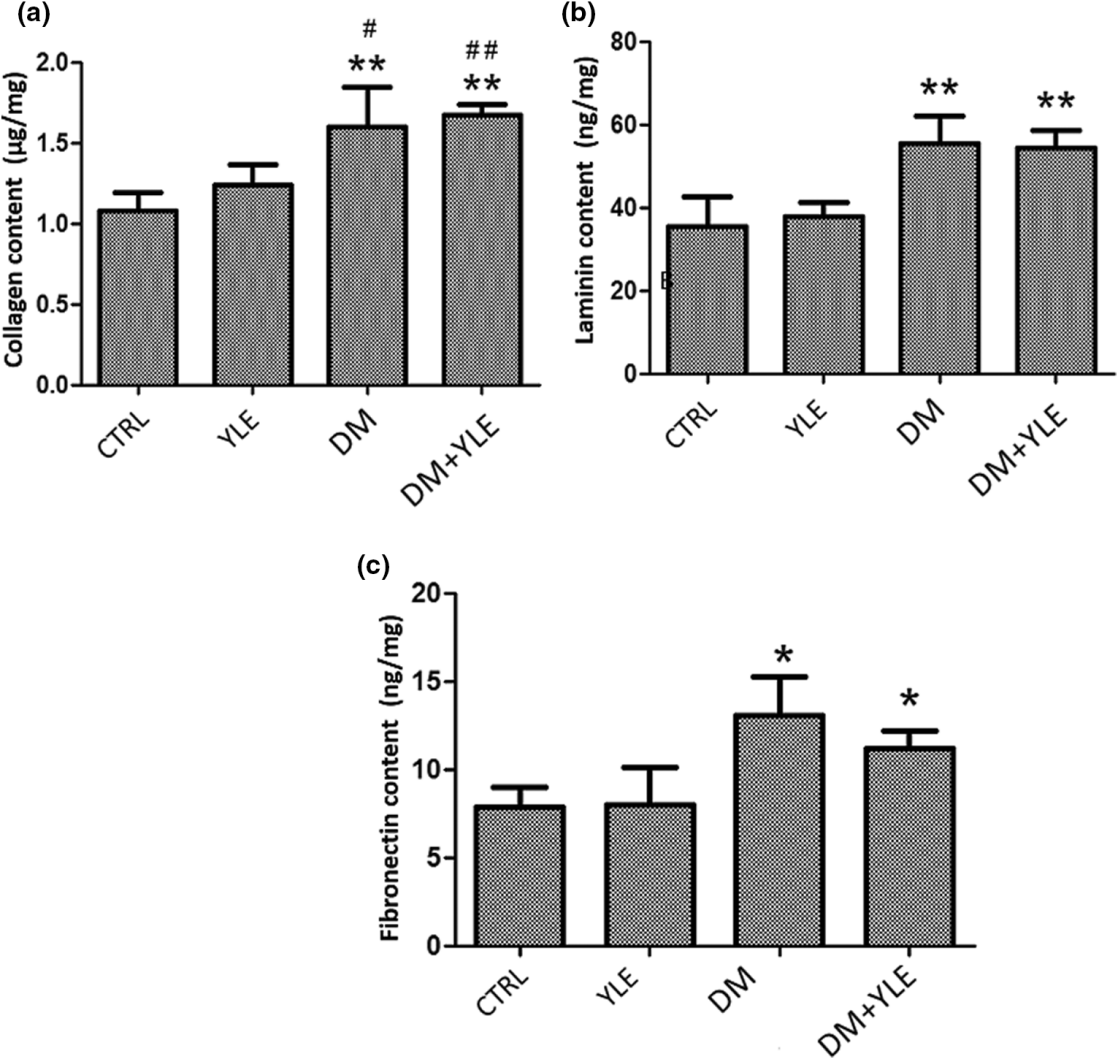
Total collagen (μg/ml) (a), laminin (ng/mg) (b), and fibronectin (ng/mg) contents (c) in the liver from control and treated rats. Data are mean ± SEM. *p < .05, **p < .01 vs. CTRL group; #*p* < .05, ##*p* < .01 vs. YLE group. CTRL, Control group; DM + YLE, Diabetic rats treated with 100 mg/kg of ethanol 50% extract of yellow lupine for 1 month; DM, Diabetic rats receiving injection of alloxan 250 mg/kg, ip; YLE, Control rats treated with 100 mg/kg of yellow lupine extract

Extracellular matrix (total collagen, laminin, and fibronectin) assay was undertaken on liver and kidney homogenates. We demonstrate that in the liver, diabetes causes a significant increase in total collagen, laminin, and fibronectin contents, respectively, by 49% (*p* < .01), 56% (*p* < .01), and 67% (*p* < .05) compared to control rats. In diabetic animals treated with yellow lupine extract, the levels of the three components of the MEC remain statistically similar to the diabetic group (Figure 1).

**FIGURE 2:**
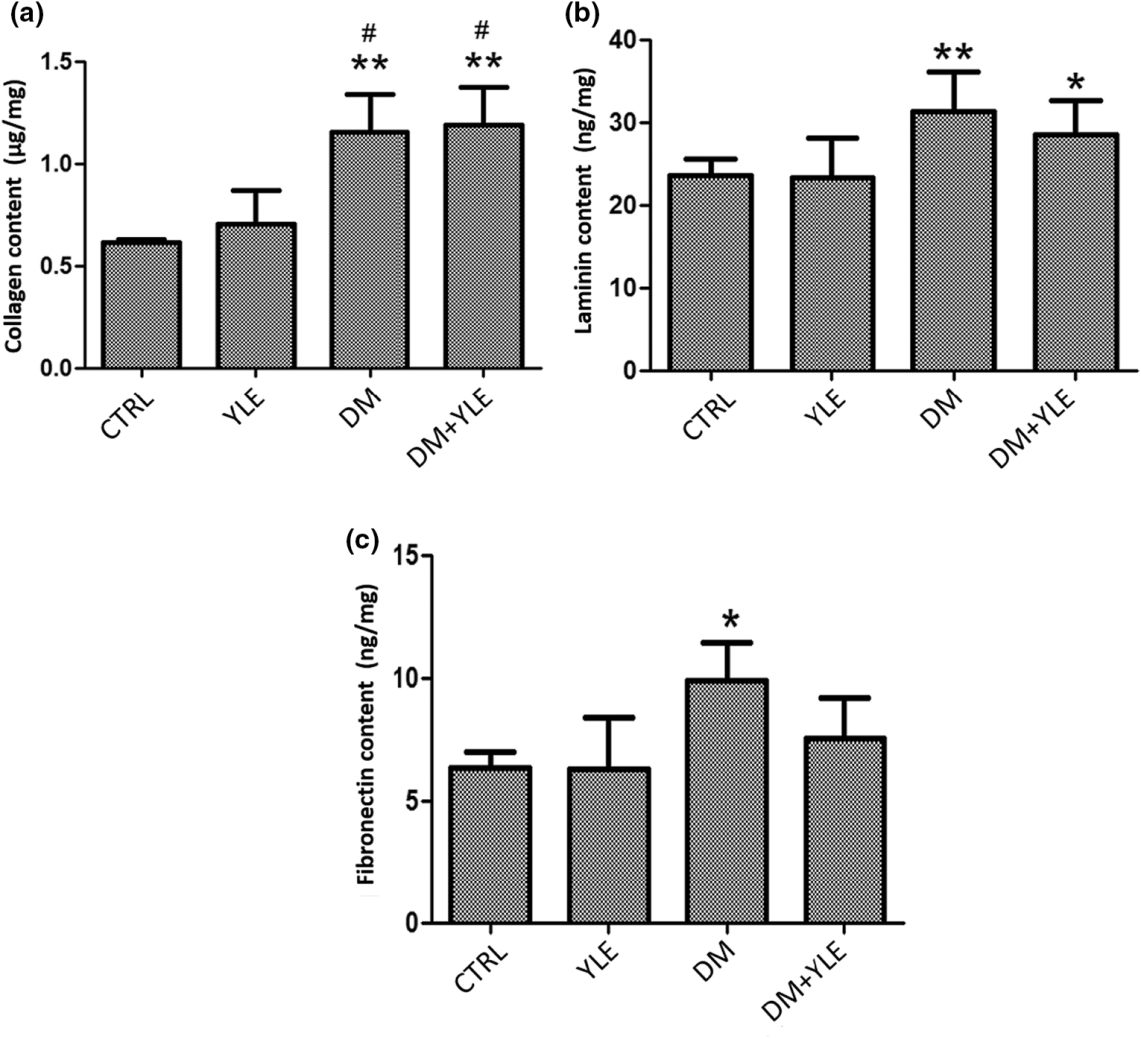
Total collagen (μg/ml) (a), laminin (ng/mg) (b), and fibronectin (ng/mg) content (c) in the kidney from control and treated rats. Data are mean ± SEM. *p < .05, **p < .01 vs. CTRL group; #*p* < .05 vs. YLE group. CTRL, Control group; DM + YLE, Diabetic rats treated with 100 mg/kg of ethanol 50% extract of yellow lupine for 1 month; DM, Diabetic rats receiving injection of alloxan 250 mg/kg, ip; YLE, Control rats treated with 100 mg/kg of yellow lupine extract.

In the kidneys, the total collagen content was increased by 91% (*p* < .01) and 88% (*p* < .01), respectively, in the rats of the DM and DM + YLE groups compared to the control rats. Similarly, the laminin content increased significantly by 35% (*p* < .01) in the DM group and by 20% (*p* < .05) in the DM + YLE group (Figure 2).

Diabetes causes a significant increase in fibronectin content by 55% in the kidneys. In the group treated with the yellow lupine extract, the laminin level remains statistically similar to the CTRL group and also to the DM group.

### 3.6 Activities of matrix metalloproteinases

**FIGURE 3:**
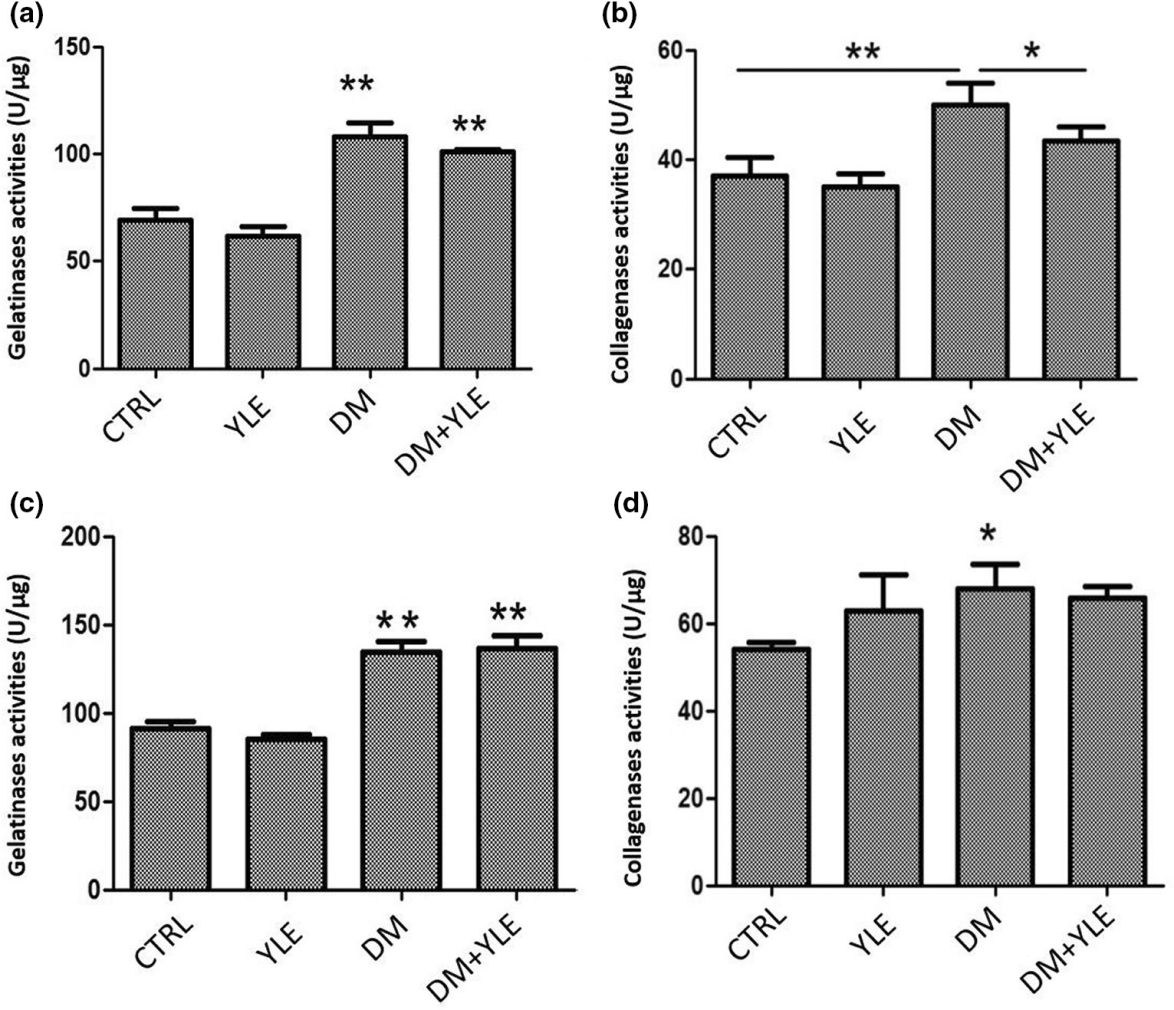
Gelatinases (MMP‐2 and MMP‐9) activities (U/μg) in liver (a) and kidney, (c) collagenases (MMP‐1, MMP‐8, and MMP‐13) (U/μg) in liver and kidney (b), and kidney (d) from control and treated rats. Data are mean ± SEM. *p < .05, **p < .01 vs. group. CTRL, Control group; DM + YLE, Diabetic rats treated with 100 mg/kg of ethanol 50% extract of yellow lupine for 1 month; DM, Diabetic rats receiving injection of alloxan 250 mg/kg, ip; YLE, Control rats treated with 100 mg/kg of yellow lupine extract

The measurement of the activity of collagenases and gelatinases (MMP‐1, MMP‐8, and MMP‐13) was undertaken on the homogenates from liver and kidney. In the liver, diabetes increases total collagenases and gelatinases activities, respectively, by 35% and 57% compared to the CTRL group. Therefore, treatment of diabetic rats with lupine extract decreased collagenases and gelatinases activities by 15% and 7%, respectively, when compared to diabetic rats (Figure 3).

In the kidneys, diabetes causes an increase in total collagenases and gelatinases activities, respectively, by 25% and 47% compared to the CTRL group. However, no significant difference was observed for collagenases and gelatinases activities between the DM and DM + YLE groups (Figure 3).

### 3.7 Histological study

**FIGURE 4:**
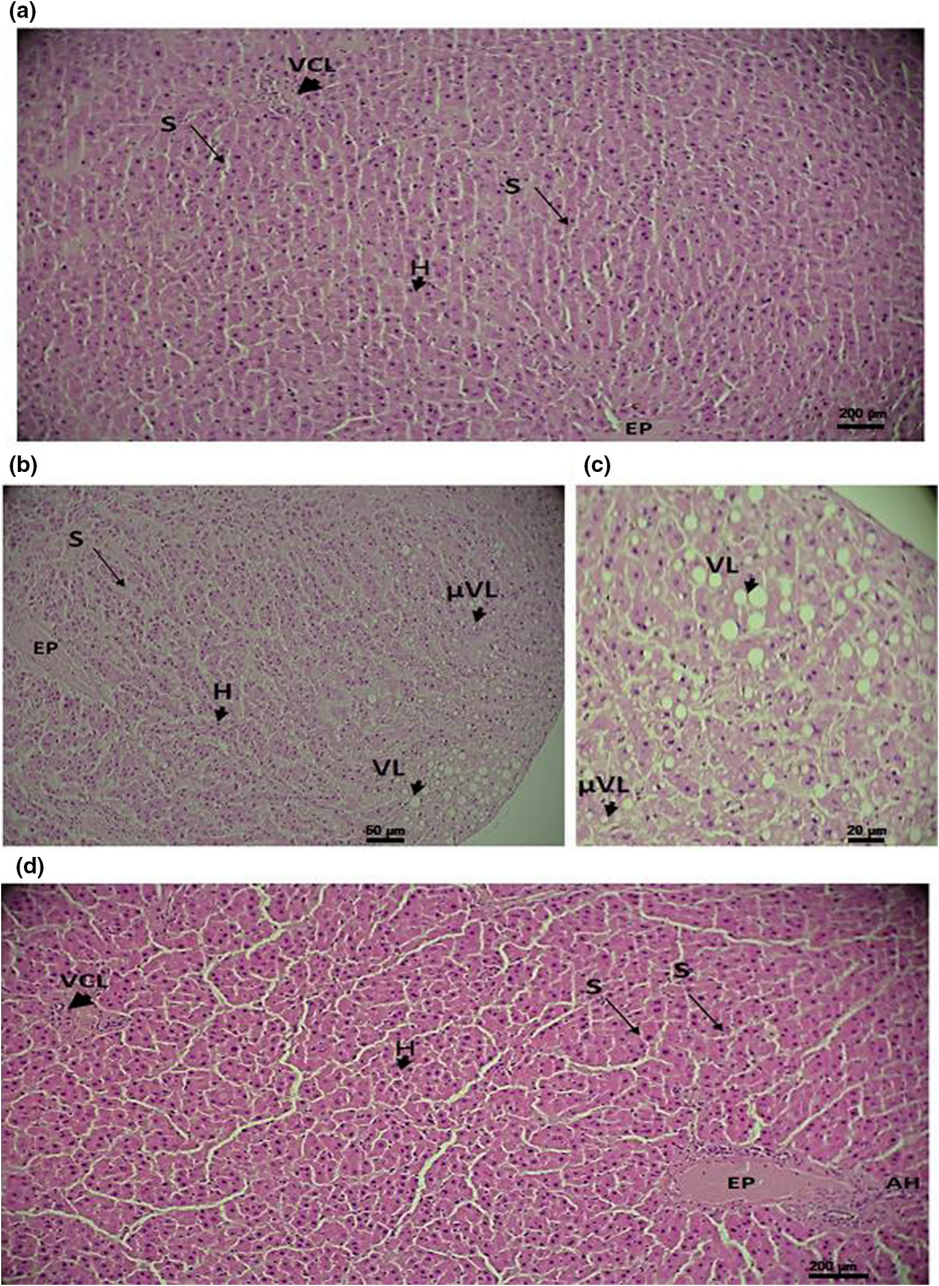
Cross‐sections of the liver stained with hematoxylin–eosin from control rats (a, G = 100×), diabetic rats by injections of alloxan 250 mg/kg (b, G = 200×; c, G = 400×), and treated with 100 mg/kg of ethanol 50% of yellow lupine for 1 month (d, G = 100×). EP, Portal area; H, Hepatocyte; HA, Hepatic artery; LV, Lipid vacuole; S, Sinusoid; VCL, Centrolobular vein; μVL, Lipid microvacuole

Histological study was carried out on cross‐sections of the liver stained with hematoxylin–eosin. In the control group and DM + YLE group, the liver had a normal appearance characterized by hepatocytes arranged in traves without any sign of necrosis or inflammatory cell infiltration. In the same group, the sinusoid veins look normal without signs of congestion (Figure 4a).

In diabetic rats, the liver shows signs of diffuse dilatation of the sinusoid veins associated with the presence of macroscopic and microscopic lipid vacuoles similar to hepatic steatosis (Figures 4b,c). However, no signs of necrosis or inflammation were observed in the diabetic group.

Figure 4d represents the appearance of the liver of diabetic rats treated with yellow lupine extract. This section shows an aspect similar to that of the controls with the absence of signs of steatosis (lipid vacuoles) and a reduction in the dilation of the sinusoids observed in the diabetic group. The appearance of hepatocytes is similar to that of controls without any sign of necrosis or inflammatory infiltrate.

**FIGURE 5:**
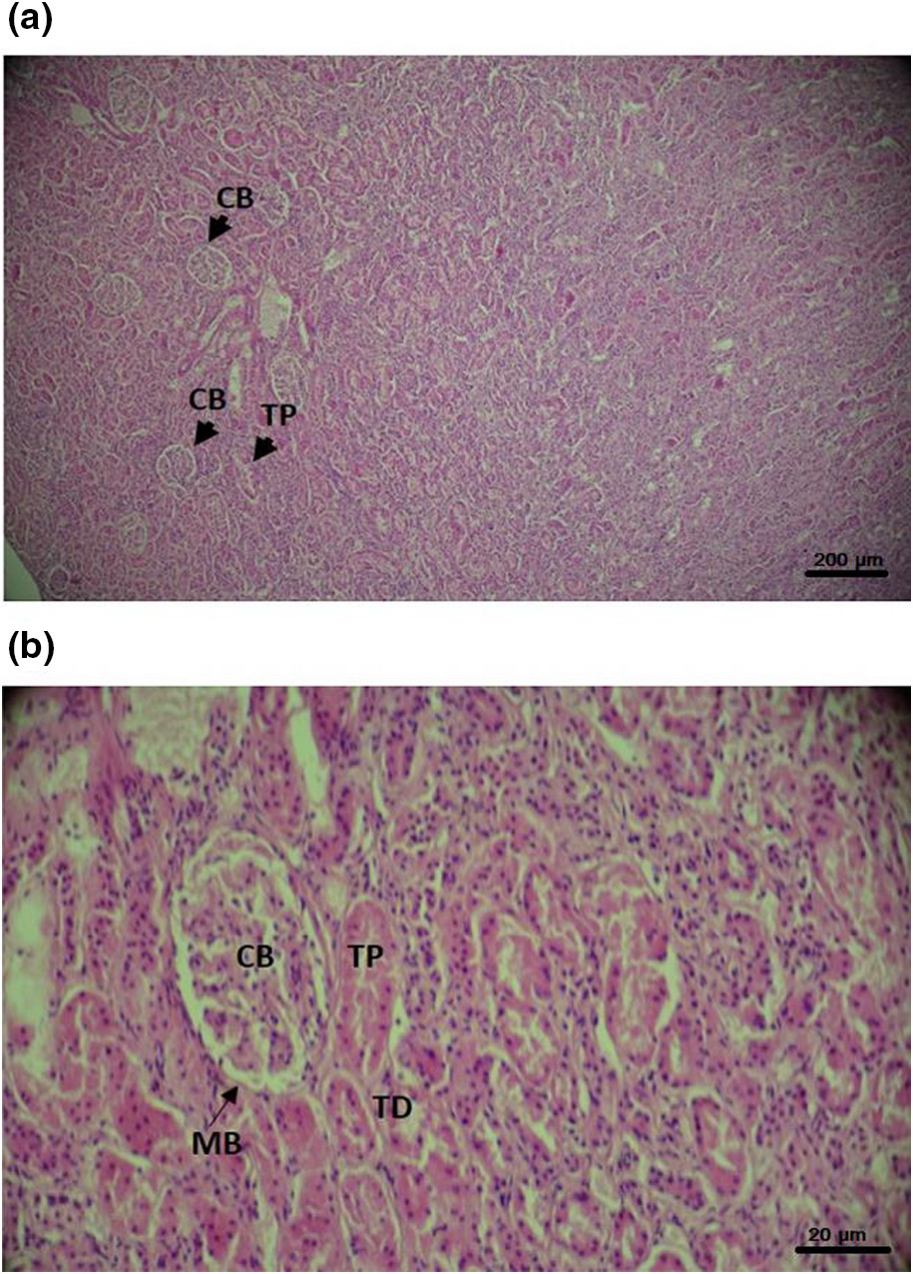
Transverse sections at kidney level stained with hematoxylin–eosin at 100× magnification (a) and 400× magnification (b). The two images are representative of the normal appearance of the kidneys similar to all used rats groups. CB, Bowman capsule; MB, Basal membrane of Bowman's cell; TD, Distal tubel; TP, Proximal tube

Histological sections from kidneys stained with hematoxylin–eosin show a normal structure of the renal tissue for the four groups of rats studied. Thus, cells appear normal in shape with no signs of inflammation or necrosis. Bowman's capsules as well as the proximal and distal tubes look normal and their basement membranes appear unaltered. No sign of fibrosis was observed (Figure 5).

## 4 DISCUSSION

In our previous study, we demonstrate that diabetes causes ECM remodeling and that some natural extracts can prevent several complications of diabetes. On the other hand, some species of lupin have been studied for their benefits on diabetes such as *Lupinus albus* and *Lupinus mutabilis* (Garmidolova et al., 2022). However, we are the first to investigate both (1) the effect of diabetes on hepatic and renal ECM remodeling and (2) the effect of yellow lupin (*L. luteus*) on experimental model of diabetes.

Our main results showed on one hand that diabetes enhances collagen, fibronectin, and laminin level as well as gelatinases and collagenases activities in kidney and liver, and on the other that lupin extract can prevent collagenases activities in diabetes rats and provide an antihyperglycemic effect and improve lipid parameters level in diabetic male rats.

In the present study, we used alloxan to induce diabetes in rats. This technique is used to obtain diabetic rats for animal experimentation purposes. We used a dose of 250 mg/kg in the form of a single intraperitoneal injection in fasting rats in accordance with the doses used previously (Dab et al., 2022). Alloxan is a structural analog of glucose, which penetrates through the glucose transporters GLUT2 of pancreatic β‐cells (Radenković et al., 2016) and causes rapid destruction of β‐cells by the simultaneous action of reactive oxygen species and massive increase in intracytosolic calcium concentration. Alloxan is thus one of the most commonly used agents for the induction of diabetes mellitus (Rohilla & Ali, 2012).

The diabetes model undertaken in our present study was successful because the blood glucose level recorded on the third day following alloxan injection exceeded 200 mg/dl. Indeed, our results show average blood glucose values of 311 mg/dl on the third day after injection of alloxan. This value reached 325 mg/dl at the end of the experiment.

The success of the model was also confirmed by the presence of polyuria diabetic rats. Moreover, we showed that diabetes was associated with an increase in cholesterol, L‐DL, and triglyceride levels in rats. Our results are consistent with previous studies in terms of the evolution of cholesterol level and triglyceride levels in human diabetes (Ahmad et al., 2008; Negreş et al., 2013; Patti et al., 2019).

Our results show that the ethanol 50% extract of yellow lupin is richer in total polyphenols than aqueous or ethanol 96% extracts. LC–MS analysis shows that *L. luteus* contains phenolic acids such as gallic acid, quinic acid, and ferulic acid in addition to flavonoids such as apigenin, quercetin, naringin, apigenin, and acacetin.

Fasting blood glucose level increases continuously in the DM group. At the end of the experiments, lupin extract significantly reduced glycemia by half compared to the DM group. However, the glycemia of the DM + YLE group remains significantly high compared to the control rats. On the other hand, the glycemia of the YLE group remains similar to that of the CTRL group.

These results confirm an antihyperglycemic effect of yellow lupine which should be used in long term. This potential antihyperglycemic action can be explained by a stimulation of insulin secretion by the remaining pancreatic β‐cells induced by the bioactive molecules in our extract. Indeed, previous studies have reported that several bioactive molecules isolated from plants such as polyphenols and flavonoids influence pancreatic β‐cells and improve insulin secretion (Chikhi et al., 2014).

Lipids play an important role in the pathogenesis of diabetes mellitus. The level of serum lipids is usually elevated in diabetes, and such an elevation represents a risk factor for coronary heart disease (Daisy & Kani, 2013). Insulin deficiency or insulin resistance is associated with symptoms of hypercholesterolemia and hypertriglyceridemia (Daisy et al., 2009). On the other hand, the treatment of rats with yellow lupine extract leads to hypolipidemia and improve triglyceride level.

We showed that AST, ALT, urea, and creatinine are similar in control and normal rats treated with lupin extract. These results confirm that yellow lupin is safe to use. On the other hand, diabetes increases significantly AST and ALT levels compared to the control diabetic group. These results are consistent with that of Abdel‐Wahhab et al. (2018) who studied transaminase levels in diabetic rats. Moreover, we confirmed impairment of renal function as demonstrated by a significant increase in uremia and creatinine level in the DM group. This tendency was reversed after the administration of lupine extract mainly for creatinine associated with a slight decrease in uremia. A previous study corroborates our observation and shows an increase in creatinine and urea levels in diabetic rats (Abdel‐Wahhab et al., 2018). The richness of our extract in secondary metabolites could explain the restoration of AST and ALT activity in the DM + YLE group compared to the DM group and corroborates our observations.

Our results show that in the liver and kidney, diabetes causes a significant increase in the content of total collagen, laminin, and fibronectin. Thus, this demonstrates a direct involvement of diabetes in the ECM remodeling in kidney and liver. Enhanced collagen level in the diabetic group was similar to previous investigations that report fibrosis installation in the lung of diabetic patients (Fariña et al., 1995). On the other hand, decreased fibroblasts adhesion, diminished response to growth factors and cytokines, and decreased production of collagens and fibronectin in wound healing were reported (Almeida et al., 2016; Hamed et al., 2010).

Laminin and fibronectin are widely present in the basal lamina which provide tissue cell‐matrix adhesion. Several pathological processes as cancers and cardiovascular disorders were associated with changes in these two glycoproteins levels (Rohwedder et al., 2012).

In the study of the activity of MMPs in the liver and kidney, diabetes caused an increase in the activity of collagenases and gelatinases. On the other hand, the administration of the yellow lupine extract to diabetic rats slightly decreases MMPs activities. MMPs cover a large family of extracellular enzymes, which share common structural features, mainly regions involved in proteolytic activity. Among these MMPs, we were interested in interstitial collagenases (MMP‐1, MMP‐8, and MMP‐13) which are the only enzymes in mammals with the ability to cut the triple helix of fibrillary collagen. Interstitial collagenase (MMP‐1) appears to have preferential activity against type III collagen. Polymorphonuclear collagenase (MMP‐8) has an affinity for type I collagen and MMP‐13 is the only collagenase able to cleave type 1, type II, and type III collagen (Malemud, 2006). MMPs activation can be occurred also by reactive oxygen species or nonphysiological agents (Loffek et al., 2011).

The histological study of liver sections stained with hematoxylin–eosin shows that in the CTRL group, the liver has a normal appearance, whereas in diabetic rats, the liver shows signs of diffuse dilation of the sinusoidal veins associated with the presence of macroscopic and microscopic lipid vacuoles which testify to a stage of hepatic steatosis. These results are consistent with the dosages of total cholesterol which have increased by diabetes.

The study of the histological sections made at the level of the kidneys and stained with hematoxylin–eosin shows a normal structure of the renal tissue for the four groups of rats studied. These observations are not consistent with the results of the assays of the ECM molecules. Indeed, it is known that ECM disorders are difficult to detect histologically in young subjects like the age of the rats used in our present study.

## 5 CONCLUSION

In this study, we first started by testing the main hypothesis that diabetes could influence the distribution of ECM components as well as the activity of MMPs at the tissue level, and secondly, by studying the systemic effects of diabetes on the status of antioxidants and the various serum parameters. We identified nine compounds by LC‐ESI‐MS in yellow lupine extract. Our main results were a significant decrease in blood glucose after treatment of diabetic rats with yellow lupine extract compared to control diabetic rats. Thus, diabetes causes a significant increase in the content of total collagen, laminin, and fibronectin in the liver and kidney. Administration of yellow lupine extract in diabetic rats caused a slight decrease in collagenase and gelatinase activities compared to diabetic rats.

## CONFLICT OF INTEREST

The authors have no conflict of interest to disclose.

## Supporting information


Supplementary material

## Data Availability

Data available in article supplementary material.
