# Supplementary material for: Effects of Lupinus luteus on hepatic and renal extracellular compounds turnover under diabetes in rat
Source: Food Sci Nutr. 2022 Dec 27;11(4):1718–27. doi: 10.1002/fsn3.3200 (PMC10084962; doi:10.1002/fsn3.3200)

**Supplementary Data**

0,0

5,0

10,0

15,0

20,0

25,0

30,0

35,0

40,0

min

1000000

1500000

2000000

2500000

3000000

3500000

4000000

4500000

5000000

5500000

6000000

6500000

2:TIC(-)

Cynarin

Gallic acid

Quinic acid

Ferulic acid

**A**

0,0

5,0

10,0

15,0

20,0

25,0

30,0

35,0

40,0

min

1000000

1500000

2000000

2500000

3000000

3500000

4000000

4500000

5000000

5500000

2:TIC(-)

Cynarin

Quinic acid

Gallic acid

Aphegenin-7-O-glucoside

Acacetin

Naringinin

Apigenin

**B**

0,0

5,0

10,0

15,0

20,0

25,0

30,0

35,0

40,0

min

1000000

2000000

3000000

4000000

5000000

6000000

7000000

8000000

9000000

2:TIC(-)

Cynarin

Quinic acid

Gallic acid

Ferulic acid

Acacetin

Aphegenin7-o-glucoside

Quercetin

Naringinin

Apigenin

**C**


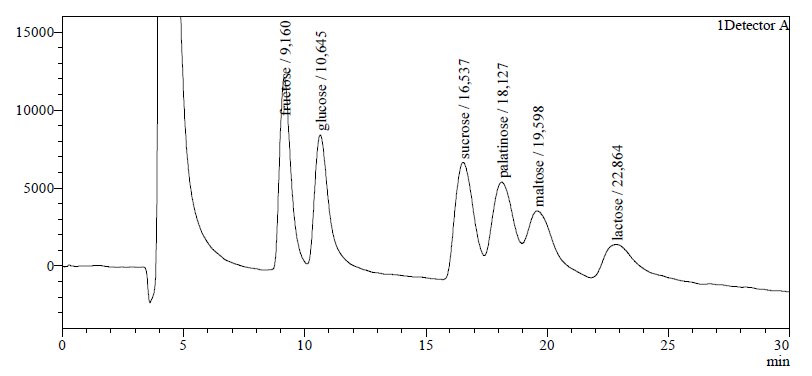

Supplement: Supplementary file 1 — Supplementary material [file FSN3-11-1718-s001.docx]
